# Duplex-Indel: a Snakemake pipeline for somatic Indel calling in Tn5 transposase-based duplex sequencing data

**DOI:** 10.1093/bioinformatics/btag205

**Published:** 2026-04-27

**Authors:** Guanlan Dong, Nazia Hilal, Shayna Mallett, Bowen Jin, Shulin Mao, Monica Devi Manam, Diane D Shao, Sangita Choudhury, August Yue Huang, Eunjung Alice Lee

**Affiliations:** Division of Genetics and Genomics, Manton Center for Orphan Disease Research, Boston Children’s Hospital, Boston, MA 02115, United States; Department of Pediatrics, Harvard Medical School, Boston, MA 02115, United States; Bioinformatics and Integrative Genomics Program, Harvard Medical School, Boston, MA 02115, United States; Division of Genetics and Genomics, Manton Center for Orphan Disease Research, Boston Children’s Hospital, Boston, MA 02115, United States; Department of Pediatrics, Harvard Medical School, Boston, MA 02115, United States; Division of Genetics and Genomics, Manton Center for Orphan Disease Research, Boston Children’s Hospital, Boston, MA 02115, United States; Department of Pediatrics, Harvard Medical School, Boston, MA 02115, United States; Division of Neuropathology, Department of Pathology, Brigham and Women’s Hospital, Harvard Medical School, Boston, MA 02115, United States; Division of Genetics and Genomics, Manton Center for Orphan Disease Research, Boston Children’s Hospital, Boston, MA 02115, United States; Department of Pediatrics, Harvard Medical School, Boston, MA 02115, United States; Program in Biological and Biomedical Sciences, Harvard Medical School, Boston, MA 02115, United States; Division of Genetics and Genomics, Manton Center for Orphan Disease Research, Boston Children’s Hospital, Boston, MA 02115, United States; Division of Genetics and Genomics, Manton Center for Orphan Disease Research, Boston Children’s Hospital, Boston, MA 02115, United States; Department of Pediatrics, Harvard Medical School, Boston, MA 02115, United States; Department of Neurology, Boston Children’s Hospital, Boston, MA 02115, United States; Division of Genetics and Genomics, Manton Center for Orphan Disease Research, Boston Children’s Hospital, Boston, MA 02115, United States; Department of Pediatrics, Harvard Medical School, Boston, MA 02115, United States; Broad Institute of MIT and Harvard, Cambridge, MA 02142, United States; Division of Genetics and Genomics, Manton Center for Orphan Disease Research, Boston Children’s Hospital, Boston, MA 02115, United States; Department of Pediatrics, Harvard Medical School, Boston, MA 02115, United States; Broad Institute of MIT and Harvard, Cambridge, MA 02142, United States; Division of Genetics and Genomics, Manton Center for Orphan Disease Research, Boston Children’s Hospital, Boston, MA 02115, United States; Department of Pediatrics, Harvard Medical School, Boston, MA 02115, United States; Broad Institute of MIT and Harvard, Cambridge, MA 02142, United States

## Abstract

**Summary:**

Duplex-Indel is a novel Snakemake workflow for detecting somatic small insertions and deletions (Indels) from Tn5 transposase-based duplex sequencing data. Duplex-Indel enhances the accuracy of mutation calling at the single-molecule level by requiring consensus support from both DNA strands for each somatic Indel, minimizing confounding from technical artifacts. Duplex-Indel extends somatic mutation calling in Tn5 transposase-based duplex sequencing data to include Indels. We have demonstrated the accuracy and robustness of Duplex-Indel using cancer cell lines.

**Availability and Implementation:**

Source code and documentation are available under the MIT license on GitHub at https://github.com/ealee-lab/duplex-indel and archived on Zenodo at https://doi.org/10.5281/zenodo.19228799.

## 1 Introduction

Somatic mutations occur spontaneously across our genomes and are consequences of DNA replication errors and DNA damage (Martincorena and Campbell 2015). As somatic mutations accumulate throughout our lifetime, they can serve as footprints of genetic and environmental factors that contribute to our understanding of aging and disease. Multiple tools have been developed for somatic mutation calling in clonal cell populations using bulk sequencing (Koboldt et al. 2012, Larson et al. 2012, Kim et al. 2018, Benjamin et al. 2019). However, detecting somatic mutations with ultra-low allele fractions, such as somatic mutations arising within post-mitotic cells, in bulk sequencing data is particularly challenging, due to the difficulty in distinguishing true mutations from the noise of sequencing errors. Single-cell whole-genome sequencing (WGS) technologies have enabled the characterization of these ultra-low-fraction mutations, but can often lead to amplification artifacts, uneven coverage, and allelic imbalance which pose challenges for accurate mutation calling (Gawad et al. 2016).

Recent advancements in duplex sequencing technologies have provided an enhanced accuracy of mutation calling through consensus calls achieved by sequencing two DNA strands independently (Abascal et al. 2021, Xing et al. 2021, Liu et al. 2024). However, existing computational tools focus on single-nucleotide variants (SNVs), neglecting other mutation types such as small insertions and deletions (Indels), which are more likely to cause frameshift and thereby disrupt gene functions (Ganz et al. 2024). META-CS (Xing et al. 2021) is a single-cell duplex sequencing method based on Tn5 transposase, where Tn5 cuts and tags the double-stranded DNA molecule with a pair of barcodes, and adaptor orientation is used to distinguish the two strands. VISTA-seq (DOI: http://dx.doi.org/10.17504/protocols.io.6qpvr3nbzvmk/v1) expands on META-CS and allows for flexible DNA input, accommodating not only single cells, but also microbulk (∼50 cells) and bulk samples. Although the existing SNV calling pipeline for META-CS can be adapted for VISTA-seq, there is no available method for Indel calling.

Furthermore, different types of duplex sequencing technologies often have distinct characteristics that require specific processing or filtering, which poses challenges in applying the mutation calling pipeline developed for one technology directly to data generated using another technology. While DupCaller (Cheng et al. 2025) claims to be applicable across barcode-based duplex technologies, it cannot accommodate variable barcode lengths, and its variant calling parameters require fine tuning to achieve a comparable performance. Indel calling is available for CODEC (Bae et al. 2023), which uses the adapter quadruplex to physically link the two DNA strands of the same molecule, and consensus can thus be formed by one read pair; however, this pipeline cannot be easily applied to Tn5-based methods, where each read pair generated contains information for only one strand. NanoSeq (Abascal et al. 2021) uses Y adapter which is more similar to Tn5-based tagging, but Indel calling is performed with customized scripts, resulting in poor adaptability. Here, we present Duplex-Indel, a novel pipeline for somatic Indel calling in Tn5 transposase-based duplex sequencing data that is compatible with multiple types of DNA input.

## 2 Pipeline description

**Figure 1:**
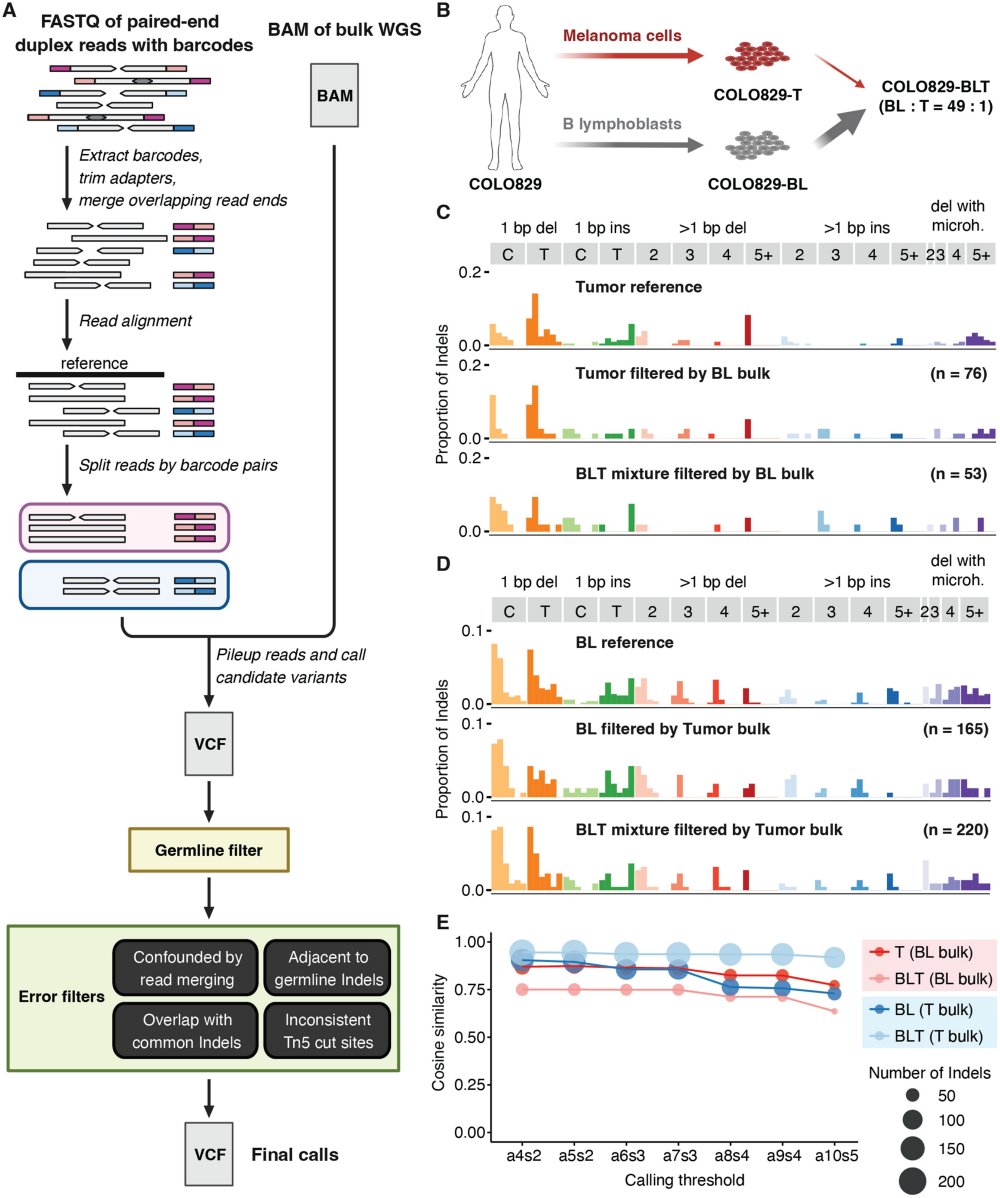
Workflow of Duplex-Indel and benchmarking analysis. (A) The pipeline takes raw reads from Tn5 transposase-based duplex sequencing data as input and requires matched bulk WGS data for removing germline variants. There are four main steps: preprocessing, duplex read alignment by barcode pairs, read pileup to generate candidate variants, and filtering to generate the final call set of somatic Indels. (B) Using cell lines derived from a melanoma patient (COLO829), we applied Duplex-Indel to duplex sequencing data generated from tumor cell line (COLO829-T), matched normal B lymphoblast cell line (COLO829-BL), and a mixture of the two cell lines (COLO829-BLT) at a ratio of COLO829-BL : COLO829-T = 49 : 1. (C) Tumor-specific Indels (filtered by BL bulk) detected by Duplex-Indel are compared to the tumor reference. Numbers of Indels are shown in parentheses. Calling threshold a4s2 was used. (D) BL-specific Indels (filtered by tumor bulk) detected by Duplex-Indel are compared to BL reference. Numbers of Indels are shown in parentheses. Calling threshold a4s2 was used. (E) Cosine similarity between the pipeline-recovered spectrum and the corresponding reference spectrum is used as benchmark to evaluate the accuracy of the pipeline. Duplex calling thresholds are shown as aXsY (e.g. a4s2), where X is the minimum number of alternative allele reads required in total, and Y is the minimum number of alternative allele reads required on each strand. The stability of cosine similarities with varying calling thresholds is used to evaluate the robustness of the pipeline. Each benchmark is indicated by color, where light and dark red represent benchmarks for tumor-specific Indels and light and dark blue represent benchmarks for BL-specific Indels. Point size represents the number of detected Indels.

Duplex-Indel utilizes the Snakemake workflow manager (Molder et al. 2021) which is scalable and easily adaptable for cluster computing. The input data include FASTQ files of short paired-end duplex sequencing reads where the ends of each read pair are tagged by a pair of barcodes, and a BAM file of matched bulk WGS data for germline variant filtering. The pipeline follows four main steps: read preprocessing, read alignment by barcode pairs, read pileup to generate candidate variants, and filtering to generate final somatic calls (Fig. 1A).

First, duplex sequencing reads are preprocessed, including extracting barcodes, trimming sequencing adapters, and merging overlapping ends of the read pair. The pipeline performs alignment on preprocessed reads using BWA-MEM (Li 2013). Aligned reads are then split by barcode pairs. Extracted barcodes are used to identify the original DNA fragment from which the reads are sequenced. Therefore, reads sharing the same barcode pair are pooled together to enable variant calling on the single-molecule level. Then, reads from both duplex sequencing and bulk WGS are piled up at each genomic position to collect reads from each sample that cover this base and calculate read counts supporting reference and alternative alleles. After removing low quality reads, candidate variants with at least four reads supporting an alternative allele are generated as an intermediate VCF file, and sex chromosomes are excluded. At this step, the pipeline can also take in a masked genome file which contains high-quality or high-confidence regions to enhance variant calling performance and reduce runtime. Indel candidates are generated using thresholds set by the number of reads supporting the alternative allele. To leverage the unique strength of duplex sequencing, we perform a complementarity check on read support from both DNA strands to produce consensus double-stranded calls. The threshold can be set using parameters “-a” and “-s.” The default calling threshold requires at least four reads supporting the alternative allele in total (a4) with at least two reads supporting the alternative allele on each strand (s2).

Finally, the pipeline applies germline and error filters to generate final somatic Indel calls. Following the germline filter which removes Indels detected in both duplex sequencing sample and bulk WGS data, the pipeline offers a series of filters to remove systematic errors and potential artifacts. Users can customize which filters to implement and how stringent each filter should be in order to obtain the most suitable combination of filters and variant calling parameters for specific applications. Read preprocessing and alignment from earlier steps of the pipeline can introduce errors that lead to false positives. During preprocessing, the overlapping ends of reads from the same read pair are merged to improve base quality and avoid confounding on read support calculation. However, this can result in false positives at tandem repeats and microhomology regions, where read ends may share a similar sequence but do not in fact overlap. During read alignment, mapping errors are more likely to occur around difficult regions such as germline Indels, also resulting in false positive calls. Therefore, filters are available to remove Indels that are either confounded by read merging or adjacent to germline Indels. While barcode pairs are good indicators for identifying reads sequenced from the same DNA molecule, a small number of available barcodes can increase the risk of barcode collision where reads sharing the same barcode pair are from different DNA molecules. To ensure variant calling is performed on the single-molecule level, we check the start and end positions of each read pair which are the cut sites of Tn5 transposase, and a filter is available to remove Indels with inconsistent Tn5 cut sites. Lastly, the pipeline can filter out common Indels with ≥ 1% population allele frequency based on the gnomAD database (Karczewski et al. 2020) to reduce potential contamination before creating a final somatic Indel call set in a VCF file.

## 3 Benchmarking on cancer cell lines

We used publicly available cell lines from a melanoma patient, COLO829, to benchmark our pipeline and evaluate our performance (Fig. 1B). In addition to the tumor cell line (COLO829-T) and the matched normal B lymphoblast (BL) cell line (COLO829-BL), we obtained a cell line mixture (COLO829-BLT) created by mixing COLO829-BL and COLO829-T at a ratio of 49 : 1 (Swanson et al. 2025), which may better simulate the low frequency of somatic mutation in real world applications. We generated duplex sequencing samples of 250 pg genomic DNA from the three samples and applied Duplex-Indel with either COLO829-BL or COLO829-T bulk WGS data for the “germline” filter to capture tumor-specific and BL-specific Indels, respectively.

First, we established the reference mutational spectra to use as “truth sets.” For tumor reference, we used the curated Indels from a previous publication (Craig et al. 2016). For BL reference, we first used Mutect2 (Benjamin et al. 2019) to call somatic Indels from bulk WGS data, with COLO829-T as the “normal” input and COLO829-BL as the “tumor” input in order to detect BL-specific variants while removing potential contamination from the tumor. We also removed germline variants of the individual and used a panel of normals to remove technical artifacts. Then, GATK FilterMutectCalls was run with default parameters, and only passing variants were retained. To obtain a high-quality call set to serve as BL reference, we further applied stringent filters: depth > 100, VAF > 0.25, excluding Indels of ≥ 1% population frequency in gnomAD (Karczewski et al. 2020), and no overlaps with difficult regions such as regions with low-mappability, low-complexity, and segmental duplications from Genome In A Bottle (GIAB) v3.5 (Olson et al. 2022).

We used cosine similarity between the reference spectrum and pipeline-recovered spectrum to evaluate the performance of Duplex-Indel, since variant-level comparison is less applicable to single-molecule-based methods like duplex sequencing, which has a high resolution to call private mutations not detectable in the bulk data but lacks full-genome coverage. The same gnomAD filter (Karczewski et al. 2020) and GIAB (Olson et al. 2022) genome mask as described above were applied to all spectra for better comparison. In addition, only autosomal Indels were considered.

Tumor-specific Indels detected from COLO829-T duplex sample (filtered by BL bulk) successfully recovered the tumor reference (cosine similarity 0.87, Fig. 1C). Despite the challenge of ∼2% presence of tumor cells in the COLO829-BLT cell line mixture, the Indel spectrum from our pipeline (filtered by BL bulk) also yielded a high similarity with the tumor reference (cosine similarity 0.75, Fig. 1C). Furthermore, BL-specific Indels from COLO829-BL duplex sample (filtered by tumor bulk) exhibited a stronger resemblance to the BL reference (cosine similarity 0.90, Fig. 1D). With a much larger presence of BL cells in the COLO829-BLT cell line mixture, BL-specific Indels detected from the mixture (filtered by tumor bulk) also yielded a high similarity with the BL reference (cosine similarity 0.95, Fig. 1D), validating the performance of our pipeline. Given the fewer number of tumor-specific Indels compared to BL-specific Indels, this suggests our pipeline is also able to accurately recover sparse spectrum. It is worth noting that, since duplex sequencing has single-molecule resolution and can detect mutations in one single cell, the slight discrepancy between our spectrum and the bulk-based truth set may result from private mutations that accumulated in BL and tumor cells during cell culture, which were missed in bulk WGS data.

We tested the robustness of the Indel spectrum recovered by Duplex-Indel by comparing it against the reference with varying calling thresholds. The cosine similarities between detected Indels from the cell lines and their respective references remain relatively high even after the threshold increases to a10s5 (Fig. 1E). At calling threshold a8s4, meaning at least eight alternative allele reads are required with at least four alternative allele reads on each strand, there is a slight drop for cosine similarities across all benchmarks except for COLO829-BLT with tumor bulk filter (light blue, Fig. 1E). This is likely due to spectrum sparsity as mentioned above, given the noticeable decrease in the number of Indels (indicated by the size of each point) across the three benchmarks (COLO829-T with BL bulk filter, COLO829-BLT with BL bulk filter, and COLO829-BL with tumor bulk filter). On the other hand, COLO829-BLT with tumor bulk filter consistently recovered more Indels and therefore demonstrated the best performance. Pipeline robustness across varying sequencing depths was also evaluated through down-sampling experiments (Fig. S1).

To assess the validity of cosine similarity as a metric, we further performed bootstrapping analysis on cosine similarities between the truth sets and biologically unrelated tissues as “negative controls.” Cosine similarities of COLO829 samples were significantly higher (above the upper bound of the 95% confidence interval) across almost all unrelated tissues (Fig. S2). In addition, we compared the performance of Duplex-Indel against DupCaller (Cheng et al. 2025) (Fig. S3). We tried two options for the noise mask–as the mask described by the authors was not available–one from NanoSeq which was similar to DupCaller’s mask, and the other from GIAB which was used by our pipeline. Otherwise, the default parameters were used. The total numbers of variants from DupCaller were higher while their cosine similarities with the truth sets were lower compared to Duplex-Indel, suggesting that additional variants from DupCaller are likely false positives. These results demonstrated a higher accuracy and better compatibility for Duplex-Indel with Tn5-based duplex data.

## 4 Discussion and conclusions

Duplex sequencing technologies have emerged to enhance mutation calling accuracy by generating consensus calls using independently sequenced DNA strands. We have developed Duplex-Indel, a Snakemake pipeline for somatic Indel calling in Tn5 transposase-based duplex sequencing data, and demonstrated its calling accuracy and robustness using melanoma cancer cell lines as benchmarks.

While well-characterized cancer cell lines are useful benchmarking data, the strength of duplex sequencing lies in ultra-low-fraction or even private somatic mutation detection which has especially been a challenge for post-mitotic tissues. Post-mitotic human tissues such as neurons accumulate somatic mutations unique to each cell’s own genome during aging, and these mutations can only be detected using single-cell or single-molecule sequencing technologies (Lodato et al. 2018, Abascal et al. 2021, Xing et al. 2021, Luquette et al. 2022). We have applied Duplex-Indel to human neurons from neurotypical controls as well as individuals diagnosed with different neurodegenerative diseases, using both single-cell (Fig. S4) and microbulk (∼50 pooled cells) input. Disease-specific Indels detected by our pipeline in human neurons have allowed us to better understand the underlying mutagenic mechanism shared across four major neurodegenerative disorders (Dong et al. 2025, Jin et al. 2025, Zhou et al. 2025). In these studies, somatic SNV calling was also performed, and more details can be found in the methods section of Dong et al.

Duplex-Indel is inspired by and expands on the SNV calling methods in META-CS (Xing et al. 2021), but it can be applied to general Tn5 transposase-based duplex sequencing data given a reference list of barcodes. While the pipeline aims to detect double-stranded Indels, single-stranded Indels can also be generated where the variant is only present on one of the two strands (Fig. S5). However, it is worth noting that there are limitations in current Tn5-based duplex sequencing methods, such that biological DNA damage cannot be directly distinguished from sequencing and library preparation artifacts among single-stranded events (Xing et al. 2021). Another method, HiDEF-Seq (Liu et al. 2024), was recently developed to detect single-stranded DNA damage with high fidelity, although it is restricted to SNVs and cannot be applied to Indels. Nevertheless, these artifacts should remain constant across samples processed under identical protocols and experimental setups, allowing true biological insight to be gained through comparative analyses, for example between disease and control groups.

The single-molecule resolution of duplex sequencing allows Duplex-Indel to accommodate both single-cell and pooled-cell input (e.g. microbulk or bulk DNA). Further, Duplex-Indel is applicable to polyploid tissues such as heart muscle, liver, and placenta, which have been difficult to study using conventional single-cell sequencing technologies (Bohrson et al. 2019, Luquette et al. 2022). These approaches rely on linkage or adjacency between germline heterozygous single-nucleotide polymorphisms (hSNPs) and somatic mutations for artifact filtering, but this linkage is disrupted in high-ploidy genomes. Our pipeline broadens the scope of somatic mutation studies and opens up the opportunities to explore the somatic mutation landscape of clonal and non-clonal populations with variable ploidy.

## Supplementary Material

btag205_Supplementary_Data

## Data Availability

Indel calls from COLO829 samples are available in Table S1 as supplementary data. Sequencing data will be shared on reasonable request to the corresponding author.
