# Supplementary material for: Duplex-Indel: a Snakemake pipeline for somatic Indel calling in Tn5 transposase-based duplex sequencing data
Source: Bioinformatics. 2026 Apr 27;42(5):btag205. doi: 10.1093/bioinformatics/btag205 (PMC13171174; doi:10.1093/bioinformatics/btag205)
Supplement: btag205_Supplementary_Data [file btag205_supplementary_data.zip › duplex_indel_supp_data_20260418.pdf]

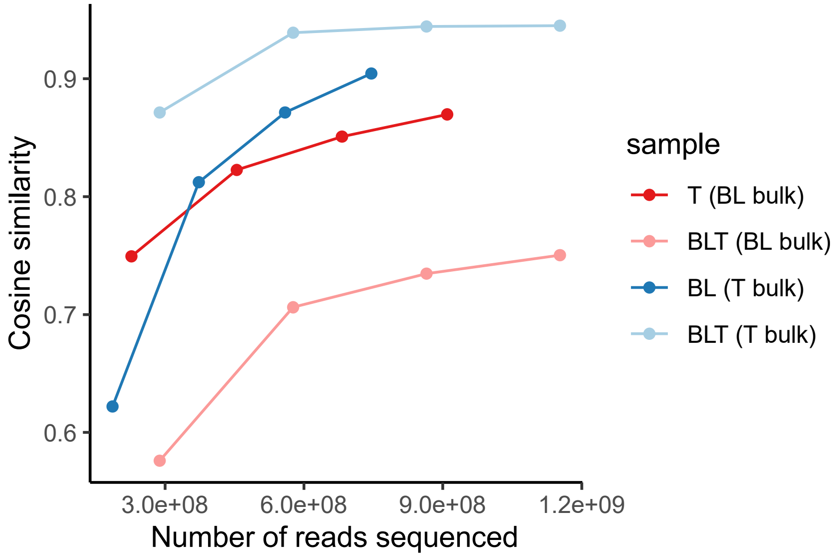

Figure S1. Pipeline robustness across varying sequencing depths. Cosine similarities between the pipeline-recovered spectrum and the corresponding reference spectrum are calculated for the original and downsampled (25%, 50%, and 75% of the original depth) samples. Sequencing depths are shown as number of reads sequenced in the FASTQ files. The stability of cosine similarities with varying sequencing depths is used to evaluate the robustness of the pipeline.

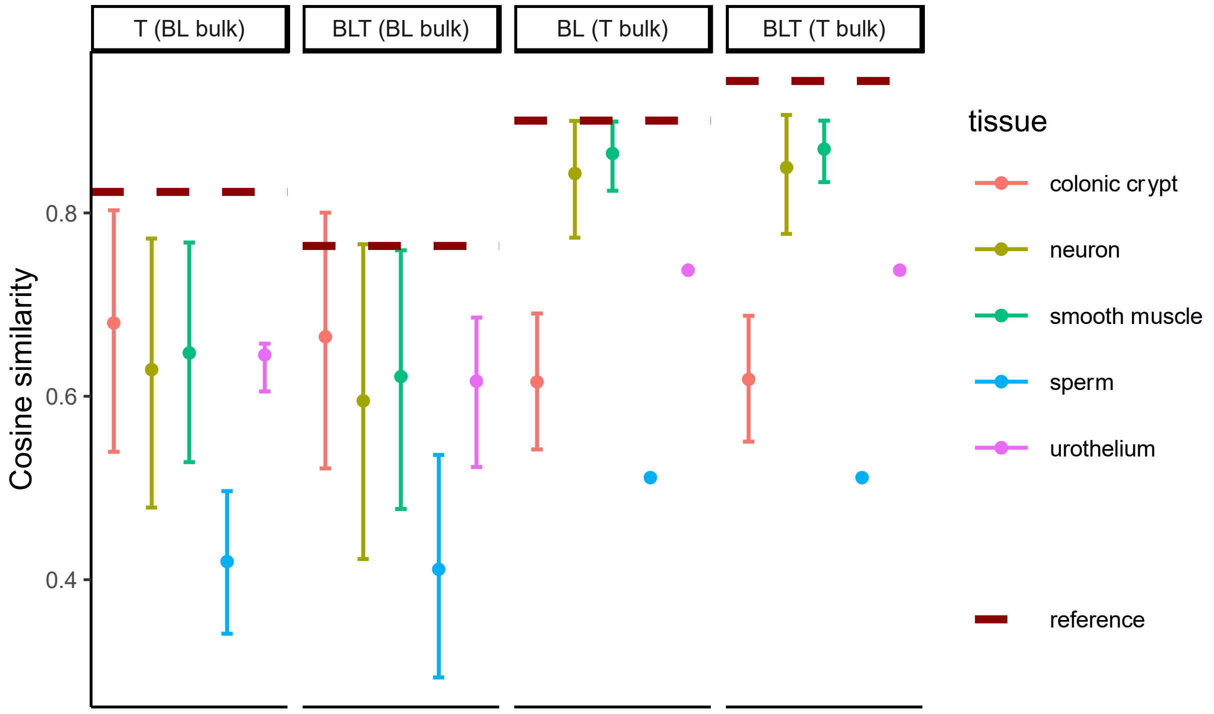

Figure S2. Cosine similarities between truth sets and unrelated tissues. Indel calls of five biologically unrelated tissue types were obtained from NanoSeq (Abascal et al. 2021). Bootstrapping (N = 1000) was used to select the same number of variants from NanoSeq call sets as each COLO829 benchmark, and a cosine similarity was calculated between each bootstrapped call set and the truth set corresponding to that benchmark. Points with error bars are the means and 95% confidence intervals (CIs) across bootstrapped cosine similarities and colored by tissue. When the original call set is smaller than the benchmark, cosine similarity was calculated using the original call set (without bootstrapping, shown as a single point). Red dashed lines indicate cosine similarities of COLO829 samples with their corresponding truth set as reference.

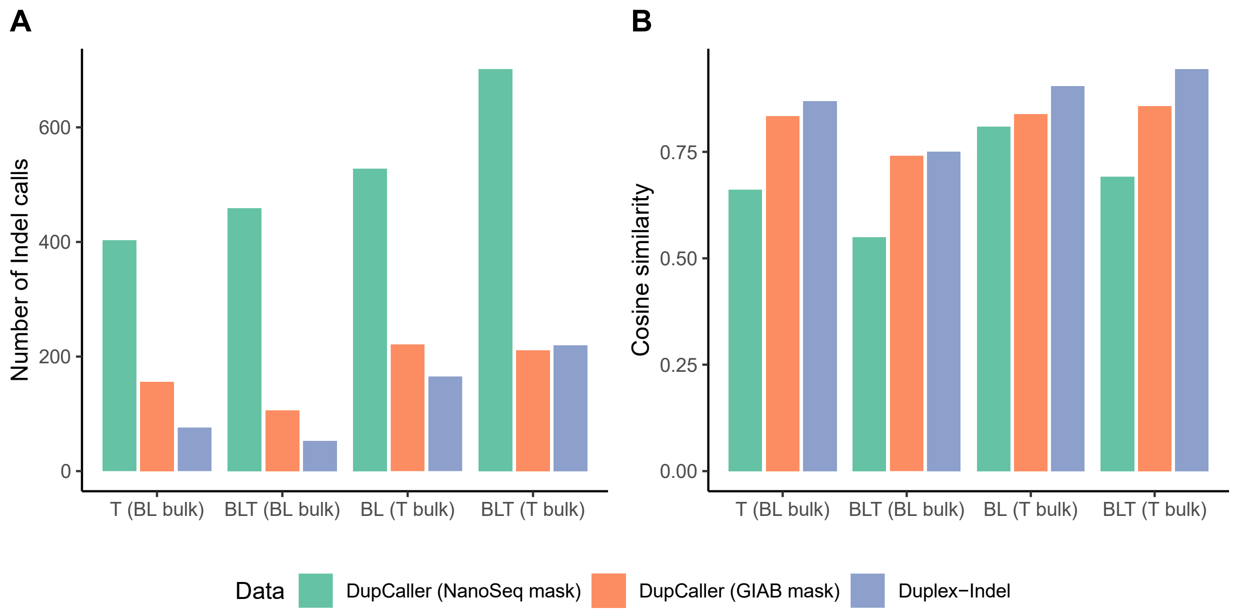

Figure S3. Comparisons between Duplex-Indel and DupCaller on COLO829 samples. DupCaller was run on COLO829 samples and their call sets were compared with those from Duplex-Indel. Noise masks from NanoSeq (similar to DupCaller's original mask which was not available) and GIAB (used by Duplex-Indel) were applied to DupCaller. **(A)** Number of variants detected by the two pipelines. **(B)** Cosine similarity between variants called by each pipeline and the corresponding truth set.

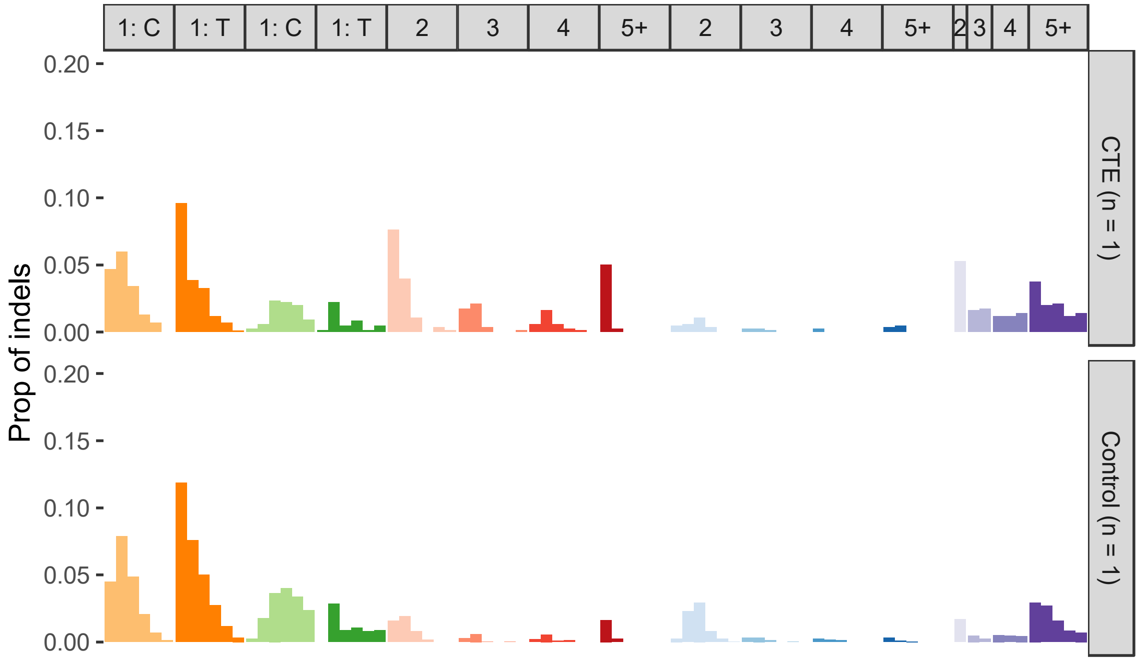

Figure S4. Application of Duplex-Indel to single neurons from CTE and neurotypical control individuals. Duplex-Indel was used to detect somatic Indels in single-cell Tn5-based duplex sequencing data. Data from Dong et al. 2025. CTE: chronic traumatic encephalopathy.

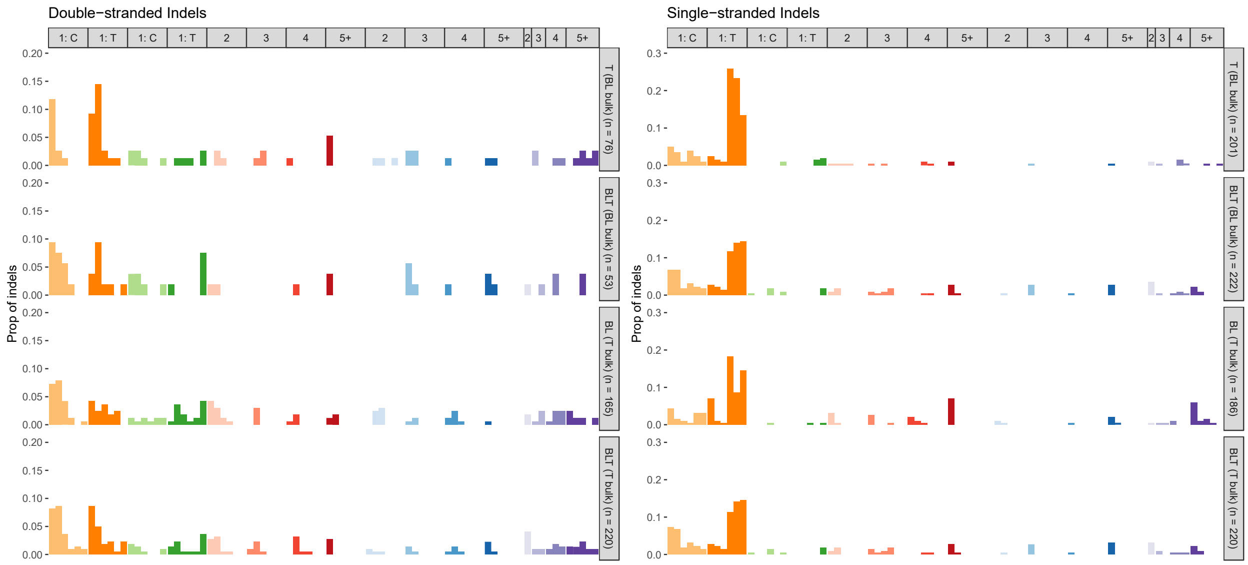

Figure S5. Double-stranded (left) and single-stranded (right) Indels detected in COLO829 samples. While the focus of Duplex-Indel is on double-stranded events, the pipeline also reports single-stranded events, though cautious interpretation is advised. Single-stranded variants are required to have 1) at least 4 alternative allele reads with no reference allele reads on the variant strand and, 2) at least 4 reference allele reads with no alternative allele reads on the nonvariant strand. Double-stranded Indels are reproduced from Figure 1 C and D.

Table S1. Indel calls in COLO829 samples. (separate file)

Table S2. Overlaps between detected variants and reference variants in COLO829 samples. Duplex-covered: genomic loci with "a4s2" coverage, i.e. at least four total reads with at least two reads from each strand.

COLO829 sample (bulk for filter)	Number of detected reference variants	Detection rate of all reference variants	Detection rate of duplex-covered reference variants
**T (BL bulk)**	41	20% (41/205)	37% (41/110)
**BL (T bulk)**	101	20% (101/512)	38% (101/267)

Experimental method. Tn5-based duplex sequencing, VISTA-seq, was used to generate the COLO829 samples used in this study. This method was adapted from META-CS (Xing et al. 2021), and a detailed experimental protocol can be found here: dx.doi.org/10.17504/protocols.io.6qpvr3nbzvmk/v1. In brief, genomic DNA was lysed and tagmented by Tn5 transposase carrying strand-specific adapters. Then, two separate rounds of strand tagging labeled each strand with a barcode, where exonuclease was used for cleanup after each round. After PCR amplification, the libraries were purified and selected for ~400-600 bp fragments. Paired-end (150 bp) sequencing was performed on Illumina NovaSeq X Plus.

Parameters in Duplex-Indel. The following parameters and settings are used in Duplex-Indel by default. During preprocessing, reads are required to have an exact match to one of the barcodes on the provided list. Both mapping quality and base quality thresholds are 20 for the bulk and 30 for the duplex sample. Alignments with clipping length at 20 bp or longer are ignored. Only autosomes are used. During calling, variant sites are required to have at least 20 reads with 0 alternative allele reads in the bulk. Variants are filtered out if they satisfy any of the following criteria: 1) having an average mapping quality (AMQ) below 50, 2) located within the masked genomic region, 3) located within 10 bp of the end of the read, 4) located within 100 bp from the previously called variant, 5) located adjacent to a germline Indel (flanking 5 bp or twice the germline Indel length, whichever is larger), 6) overlapping with any provided common variants in the population. In addition, the following parameters are made accessible for the user in variant calling. -a, minimum number of alternative allele reads in total. -s, minimum number of alternative allele reads on each strand. -R, maximum number of reference allele reads allowed. -B, minimum alternative allele balance at the single-molecule level (requires -R to be > 0). -S, minimum number of reads on each strand for single-stranded events, where no reference allele reads are on the variant strand and no alternative allele reads are on the nonvariant strand. -T, read merging window filter stringency, where three levels are available: -T1 filters variants with the start and end positions exactly matching the read merging window (variant start position = merging window start position, variant end position = merging window end position); -T2 filters variants with the start and end positions within 2 bp of the read merging window (variant start position within flanking 2 bp of the merging window start position, and vice versa for the end position); -T3 filters variants with any overlaps with the read merging window.

Benchmarking analysis. After following the experimental protocol, paired-end reads were obtained for three COLO829 samples, COLO829-T, COLO829-BL, and COLO829-BLT. Bulk WGS BAM files for COLO829-T and COLO829-BL were obtained from the SMaHT consortium. Duplex-Indel (v1.0.1) was configured for each benchmark based on the example file provided on the Github (https://github.com/ealee-lab/duplex-indel/blob/main/scripts/config_hg38.yaml), using reference genome GRCh38, non-difficult regions from Genome In A Bottle (GIAB) v3.5 (Olson et al. 2022), and common Indels with ≥ 1% allele frequency in gnomAD (Karczewski et al. 2020). The bulk BAM file was set to either COLO829-T or COLO829-BL depending on the benchmark. The pipeline run used the following parameters: -a4 -s2 -R0 -S4 -T1, where -a4 requires at least 4 alternative allele reads in total, -s2 requires at least 2 alternative allele reads on each strand, -R0 requires no reference allele reads, -S4 requires (for single-stranded events) at least 4 alternative allele reads with no reference allele reads on the variant strand and at least 4 reference allele reads with no alternative allele reads on the nonvariant strand, and -T1 removes variants with an exact overlap to the read merging

window. Then, a post-calling filter was applied, by setting parameter -m in apply_filters.sh, to further remove variants confounded by read merging using unmerged BAM. Calling was restricted to autosomes. Reference variants serving as the "truth set" for COLO829-T were obtained from a previous publication (Craig et al. 2016) and liftover from GRCh37 to GRCh38 using Picard (v2.8.0) LiftoverVcf. Reference variants serving as the "truth set" for COLO829-BL were obtained by running Mutect2 on bulk WGS BAM files from COLO829-T and COLO829-BL, where COLO829-T was set as "-normal". A list of germline variants from COLO829-BL was used for "--germline-resource", and a GATK panel of normal (gs://gatk-best-practices/somatic-hg38/1000g_pon.hg38.vcf.gz) was used for "--panel-of-normals". Then, GATK FilterMutectCalls was run on the VCF output with default parameters to keep only PASS variants. For consistent comparisons with the duplex calling results, non-autosomal variants and variants that overlap with the same genome mask from GIAB or common variants from gnomAD were further removed from the "truth sets". Finally, cosine similarities of COLO829-T reference with detected variants from duplex sample COLO829-T with BL bulk filter and those from duplex sample COLO829-BLT with BL bulk filter were computed to assess tumor-specific Indels; cosine similarities of COLO829-BL reference with detected variants from duplex sample COLO829-BL with T bulk filter and those from duplex sample COLO829-BLT with T bulk filter were computed to assess BL-specific Indels.

Benchmarking analysis with DupCaller. We performed additional benchmarking of DupCaller on COLO829 samples. DupCaller was downloaded and installed following instructions on the Github repository (https://github.com/AlexandrovLab/DupCaller/tree/main, commit hash 918572d). Raw FASTQ files from COLO829 samples were used as input data for barcode trimming. DupCaller only allows a fixed length of barcode, but since VISTA-seq's barcodes are of different lengths, we used the most common length. Read alignment and MarkDuplicates were performed using the specified command lines. Default parameters and the following input files were used for variant calling (same as the ones used for Duplex-Indel): GRCh38 human reference genome, bulk WGS data as matched normals, and gnomAD data as germline VCF. For the noise mask, the authors mentioned they generated a mask combining the original one from NanoSeq and satellite regions near the centromeres, but such noise mask was not provided with the preprint or on the Github repository. Therefore, we tried two options for the noise mask, one from NanoSeq which was a proxy to DupCaller's mask, and the other from GIAB which was used by our pipeline.

Downsampling analysis. To evaluate the robustness of performance across varying sequencing depths, COLO829 samples were downsampled at a series of proportions (25%, 50%, and 75%) of the original depth. We ran "seqtk sample" on each FASTQ file with parameters 0.25, 0.5 and 0.75. Then, the pipeline was run on the downsampled FASTQ files in the exact same manner with the same parameters and filters as described above. Sequencing depths were computed as number of reads sequenced from the original and downsampled FASTQ files.

Bootstrapping analysis on unrelated tissues. Indel calls of five biologically unrelated tissue types were obtained from NanoSeq (Abascal et al. 2021). For each tissue, bootstrapping (N = 1000) was used to select the same number of variants from NanoSeq call sets as each COLO829 benchmark, and a cosine similarity was calculated between each bootstrapped call set and the truth set corresponding to that benchmark. Then, the mean and 95% confidence intervals (CIs)

across bootstrapped cosine similarities were computed for each tissue for each COLO829 benchmark. Original call sets from sperm and urothelium are smaller than the two BL benchmarks (BLT with T bulk and BL with T bulk), so no bootstrapping was done, and cosine similarities were calculated using their original call sets.

Runtime and computational resources. The average runtime of COLO829 samples was 23 hours. Computing was performed on an HPC cluster using the SLURM system, where the maximum per-job resource usage was 16.5 GB of memory and 16 CPU cores.
